# Health-Related Quality of Life after Dengue Fever, Morelos, Mexico, 2016–2017

**DOI:** 10.3201/eid2604.190729

**Published:** 2020-04

**Authors:** Annika Schulte, Ingo Weber, Diana Carolina Tiga-Loza, Irma Y. Amaya Larios, Donald S. Shepard, Cynthia A. Tschampl, Eduardo A. Undurraga, Ruth A. Martínez-Vega, Florian Fischer, Lilia Chihu, Jose Ramos-Castañeda

**Affiliations:** Bielefeld University, Bielefeld, Germany (A. Schulte, I. Weber, F. Fischer);; Universidad Manuela Beltrán, Bucaramanga, Colombia (D.C. Tiga-Loza);; National Institute of Public Health, Cuernavaca, Mexico (I.Y. Amaya Larios, L. Chihu, J. Ramos-Castañeda);; Brandeis University, Waltham, Massachusetts, USA (D.S. Shepard, C.A. Tschampl);; Pontificia Universidad Catolica de Chile, Santiago, Chile (E.A. Undurraga);; Núcleo Milenio para el Estudio del Curso de Vida y la Vulnerabilidad, Santiago (E.A. Undurraga);; Universidad Industrial de Santander, Bucaramanga (R.A. Martinez-Vega);; Universidad Anahuac, Huizquilican, Mexico (J. Ramos-Castañeda)

**Keywords:** Dengue fever, arbovirus infection, arboviruses, viruses, quality of life, health-related quality of life, HRQOL, health status, life quality, persistent symptoms, economic burden, burden of disease, Mexico, vector-borne infections, dengue virus

## Abstract

We adapted the EQ-5D-3L questionnaire and visual analog scale to assess health-related quality of life (HRQOL) and persistent symptoms in 79 patients with laboratory-confirmed dengue in Morelos, Mexico. The lowest HRQOLs were 0.53 and 38.1 (febrile phase). Patients recovered baseline HRQOL in ≈2 months.

Each year, up to 400 million dengue virus (DENV) infections and ≈40,000 deaths occur globally, costing ≈US $9 billion (*1**–**3*). Accurate estimates of disease are needed to track health progress, evaluate prevention and control technologies, and define research priorities (*4*). However, substantial heterogeneity exists in estimates of disease severity and sequelae (*5*). Research suggests dengue symptoms may persist well beyond the acute febrile phase in some patients (*6**–**8*). Little is known about health-related quality of life (HRQOL) for dengue (*7*,*8*). Despite acknowledgement of symptom persistence since 1997 (*9*), most studies focus on the febrile phase, probably substantially underestimating long-term effects of dengue (*2*,*3*,*6*). We investigated HRQOL of dengue patients during their entire laboratory-confirmed dengue episode.

## The Study

All study participants signed informed consent forms. The Ethics Committee of the National Institute of Public Health (project nos. 1223, 1755) approved the study.

We recruited participants with dengue from inpatient and outpatient facilities in Morelos, Mexico, during 2016–2017. Inclusion criteria were age >18 years, visit to a healthcare facility 2–6 days after fever onset, laboratory confirmation of DENV infection, permanent residence in Morelos, and a landline telephone. We excluded patients with cognitive impairment, psychiatric diagnoses, specific chronic diseases, and pregnancy. The final sample comprised 79 patients (Appendix Table 1).

Participants underwent a face-to-face questionnaire interview during the febrile phase and were contacted for follow-up regularly for 1 month. After 1 month, participants were contacted until they did not have dengue symptoms or until 6 months after fever onset (Appendix Table 2). Thus, estimates of HRQOL after 1 month constituted only patients with persistent symptoms.

We used an adapted version of a 3-level EQ-5D (EQ-5D-3L) instrument, a standardized method for measuring health status, to measure patients’ HRQOL (*10*), including a visual analog scale (EQ-VAS) to estimate self-reported health status. The EQ-5D-3L questionnaire collects information about patient quality of life in 5 health domains: mobility, self-care, usual activities, pain/discomfort, and anxiety/depression. We also measured quality of life using the EQ-VAS scale (0–100, worst to best health). We then created a single EQ-5D-3L index value for the HRQOL (0–1, worst to best health; Appendix Table 4) (*11*). We divided time into day-ranges (0–6, 7–15, 16–30, 31–60, 61–120, and 121–180) because not all participants responded to the questionnaires on the exact same days.

We analyzed changes in HRQOL over time using survival and Cox regression analyses. We defined recovery as baseline HRQOL (before DENV infection) and calculated the time it took each patient to recover. We estimated HRQOL recovery time for subgroups of patients using Kaplan-Meier with log-rank test statistic and identified significant predictors of HRQOL using Cox regression analyses.

The final sample comprised 62% ambulatory and 38% hospitalized patients. Most participants (retrospectively) reported no symptoms before dengue onset. The most affected domains were pain/discomfort, usual activities, and mobility. Almost all participants reported some/extreme problems during the first 6 days (92% pain/discomfort, 87% usual activities, 80% mobility). The proportion of participants reporting problems in any domain increased at 7–15 days after fever onset and remained largely stable until day 30 (Table 1). Among sampled patients, 56% reported dengue-related symptoms >30 days; 48%, >1 severe symptom; and 73%, >1 warning sign. Participants needed an average of 46.7 days to completely recover their baseline HRQOL.

**Table 1:** Patients with laboratory-confirmed dengue who reported some or extreme problems during the first 30 days after onset of dengue fever symptoms, Morelos, Mexico, 2016–2017*

EQ-5D-3L dimension	No. (%) patients, N = 79			
	Before fever, n = 77	1–6 d, n = 79	7–15 d, n = 71	16−30 d, n = 74
Mobility	1 (1.3)	63 (79.7)	57 (80.3)	59 (79.7)
Self-care	0	43 (54.4)	39 (54.9)	42 (56.8)
Usual activities	2 (2.5)	69 (87.3)	65 (91.5)	65 (87.8)
Pain/discomfort	2 (2.5)	73 (92.4)	66 (93)	63 (85.1)
Anxiety/depression	4 (5.1)	27 (34.2)	28 (39.4)	30 (40.5)

*Patients were >18 years of age. n values indicate number of patients responding to questionnaire during the indicated day range. Health-related quality of life was assessed by an adapted EQ-5D-3L questionnaire (Appendix Table 3) for reporting of problems after 1 month since fever onset (i.e., days 31–60, 61–120, 121–180).

**Figure 1:**
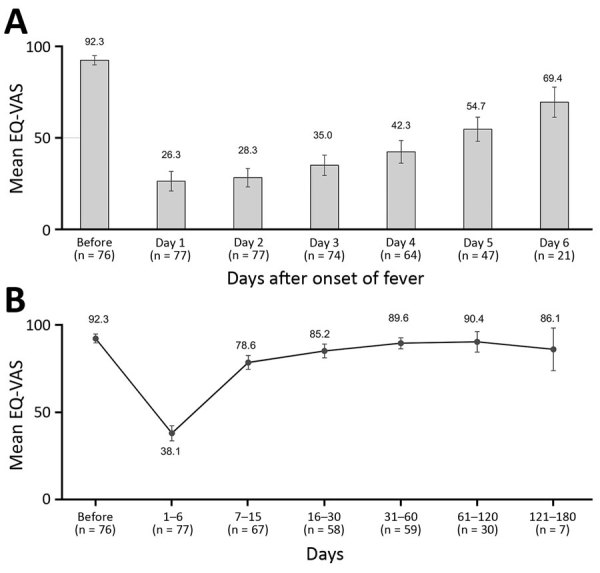
Average self-reported health status, as measured by a 0–100 EQ-VAS, of patients with laboratory-confirmed dengue during the first week after onset of dengue symptoms (A) and from baseline to 121–180 days (B), Morelos, Mexico, 2016–2017. The EQ-VAS scale measures self-reported health, ranging from 0 (worst health status) to 100 (best health status). EQ-VAS is part of the EQ-5D-3L instrument for measuring health-related quality of life. EQ-VAS, visual analog scale. n values indicate number of patients responding to questionnaire during the indicated day range. Error bars indicate 95% CI.

We also assessed participants’ self-reported health status (EQ-VAS) during the first 6 days (Figure 1, panel A). Participants reported good health at baseline (EQ-VAS 92.3 [95% CI 89.8–94.8]). The worst health was reported during the first day (EQ-VAS 26.3 [95% CI 20.9–31.7]) and second day (EQ-VAS 28.3 [95% CI 23.2–33.3]) and slowly improved until day 6 (EQ-VAS 69.4 [95% CI 61.2–77.7]) but remained well below baseline. When we analyzed the evolution of perceived health until the end of the study (Figure 1, panel B), mean EQ-VAS was 38.1 (95% CI 33.8–42.5) for days 1–6, the lowest observed for any day range. The mean EQ-VAS score then improved until days 61–120 (EQ-VAS 90.4 [95% CI 84.5–96.3]), when it no longer differed significantly from baseline (α = 0.05).

**Figure 2:**
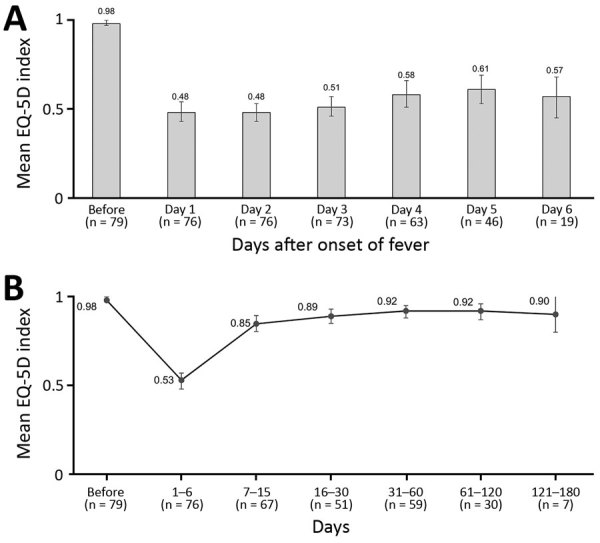
Average health-related quality of life, as measured by the EQ-5D index score, in patients with laboratory-confirmed dengue during days 1–6 of a dengue fever episode (A) and from baseline to 121–180 days (B), Morelos, Mexico, 2016–2017. The EQ-5D scale is a standardized method for measuring health status. n values indicate number of patients responding to questionnaire during the indicated day range. Error bars indicate 95% CI.

We assessed the mean EQ-5D index score before DENV infection (baseline) and during the first 6 days of illness (Figure 2, panel A). Participants showed high baseline scores (EQ-VAS 0.98 [95% CI 0.96–0.99]). The mean EQ-5D index score dropped by >50% to 0.48 (95% CI 0.42–0.49) during the first day and was 0.57 (95% CI 0.46–0.69) on day 6. During the course of the study period, the EQ-VAS was low during the first 6 days (0.53 [95% CI 49–0.58]) and increased to 0.85 (95% CI 0.80–0.89) for days 7–15 (Figure 2, panel B). The index EQ-VAS did not differ significantly from baseline after ≈61 days (0.92 [95% CI 0.88–0.98]).

We tested differences in HRQOL recovery time using Kaplan-Meier curves for individual subgroups (Appendix Figure 1). Survival curves showed slower recovery times for hospitalized participants (≈40% recovered baseline HRQOL) than for ambulatory participants (≈75%) after 30 days (p = 0.012). Participants with severe symptoms (≈30%) also showed slower recovery than did participants without severe symptoms (≈75%) after 20 days (p = 0.001), as did participants with >1 warning signs (≈40%) compared with participants without warning signs (≈85%) after 15 days (p<0.001). Participants with higher education had a faster recovery of HRQOL than did participants with less education (p<0.001).

We used a Cox regression analysis (Table 2) to identify factors associated with HRQOL recovery (model: proportionality confirmed; mean variance inflation factor = 1.09, all variables variance inflation factor <1.21; final model χ^2^ 37.8, p<0.001; McFadden pseudo-R^2^ = 0.11). Recovery rates were higher for men than for women (hazard ratio [HR] 1.87; p = 0.036), patients with more education (HR 2.06; p = 0.042), and patients with no severe symptoms (HR 2.82; p = 0.001). In the first 15 days of disease, dengue patients without skin ache had a 63% lower likelihood (HR 0.37; p = 0.002) and patients without scaling had a 67% lower likelihood (HR 0.33, p = 0.038) of recovering to baseline HRQOL.

**Table 2:** Results of the Cox regression analysis to identify factors associated with recovering baseline health-related quality of life, Morelos, Mexico, 2016–2017

Factor	Hazard ratio (95% CI)	p value
Sex		
F	Referent	
M	1.87 (1.04–3.37)	0.036
Age, y		
>38	Referent	
18–37	1.74 (0.93–3.23)	0.082
Educational level		
Primary/secondary school	Referent	
High school or higher	2.06 (1.03–4.11)	0.042
Symptoms		
Severe symptoms		
Presence	Referent	
Absence of >1	2.82 (1.50–5.33)	0.001
Persistence of symptoms		
No persistence	Referent	
Persistence <30 d	2.28 (1.24–4.19)	0.008
Specific symptoms in the first 15 d		
Presence of specific symptom	Referent	
Absence of skin ache	0.37 (0.19–0.70)	0.002
Absence of scaling skin	0.33 (0.11–0.94)	0.038
Absence of abdominal pain	1.65 (0.79–3.44)	0.182

## Conclusions

Dengue significantly reduces HRQOL beyond the febrile phase. Mobility, pain, and usual activities were the most affected domains, consistent with previous studies (*8*,*12*). The proportion of patients reporting problems remained stable among patients with persistent symptoms of dengue. HRQOL decreased abruptly during the febrile phase; most patients then steadily recovered, with some exceptions for those who had not reached baseline HRQOL at 6 months. Other studies have found larger reductions of HRQOL than we found; mean EQ-VAS score was 7 for children 0–14 years of age in Cambodia (*13*) and 10 for hospitalized patients and 20 for ambulatory patients in Brazil (*7*). Our findings were comparable to those of Armien et al. (*14*) in Panama (EQ-VAS 35.2 for children; 31.9 for adults). Female sex was significantly associated with dengue severity in our study, and education (a proxy for socioeconomic status) might be a protective factor. We found skin symptoms to be associated with a faster recovery, possibly because of a lower inflammatory or immune response (*15*).

Our findings are subject to limitations: an adults-only sample; limited socioeconomic characterization of participants; lack of data about previous DENV infections; limitations of the EQ-5D-3L instrument; possible recall bias for baseline HRQOL; response-, recalibration-, and reconceptualization response–shift biases; and a relatively small sample of patients with laboratory-confirmed dengue. Despite these limitations, our findings are relevant for clinical practice and health services research and can help researchers and other stakeholders improve estimates of dengue effects.

Appendix Additional methods and results for study of health-related quality of life after dengue fever, Morelos, Mexico, 2016–2017.

## References

[1] Bhatt S, Gething PW, Brady OJ, Messina JP, Farlow AW, Moyes CL, et al. The global distribution and burden of dengue. Nature. 2013;496:504–7. 10.1038/nature12060 23563266 PMC3651993

[2] Shepard DS, Undurraga EA, Halasa YA, Stanaway JD. The global economic burden of dengue: a systematic analysis. Lancet Infect Dis. 2016;16:935–41. 10.1016/S1473-3099(16)00146-8 27091092

[3] Stanaway JD, Shepard DS, Undurraga EA, Halasa YA, Coffeng LE, Brady OJ, et al. The global burden of dengue: an analysis from the Global Burden of Disease Study 2013. Lancet Infect Dis. 2016;16:712–23. 10.1016/S1473-3099(16)00026-8 26874619 PMC5012511

[4] Chan M, Kazatchkine M, Lob-Levyt J, Obaid T, Schweizer J, Sidibe M, et al. Meeting the demand for results and accountability: a call for action on health data from eight global health agencies. PLoS Med. 2010;7:e1000223. 10.1371/journal.pmed.1000223 20126260 PMC2811154

[5] Hung TM, Clapham HE, Bettis AA, Cuong HQ, Thwaites GE, Wills BA, et al. The estimates of the health and economic burden of dengue in Vietnam. Trends Parasitol. 2018;34:904–18. 10.1016/j.pt.2018.07.007 30100203 PMC6192036

[6] Tiga DC, Undurraga EA, Ramos-Castañeda J, Martínez-Vega RA, Tschampl CA, Shepard DS. Persistent symptoms of dengue: estimates of the incremental disease and economic burden in Mexico. Am J Trop Med Hyg. 2016;94:1085–9. 10.4269/ajtmh.15-0896 26976885 PMC4856607

[7] Martelli CMT, Nascimento NE, Suaya JA, Siqueira JB Jr, Souza WV, Turchi MD, et al. Quality of life among adults with confirmed dengue in Brazil. Am J Trop Med Hyg. 2011;85:732–8. 10.4269/ajtmh.2011.11-0067 21976580 PMC3183785

[8] Lum LCS, Suaya JA, Tan LH, Sah BK, Shepard DS. Quality of life of dengue patients. Am J Trop Med Hyg. 2008;78:862–7. 10.4269/ajtmh.2008.78.862 18541760

[9] World Health Organization. Dengue haemorrhagic fever: diagnosis, treatment, prevention and control. 2nd ed. Geneva: The Organization; 1997.

[10] EuroQol Group. EuroQol—a new facility for the measurement of health-related quality of life. Health Policy. 1990;16:199–208. 10.1016/0168-8510(90)90421-9 10109801

[11] Zarate V, Kind P, Chuang LH. Hispanic valuation of the EQ-5D health states: a social value set for Latin Americans. Value Health. 2008;11:1170–7. 10.1111/j.1524-4733.2008.00349.x 18489516

[12] Tran BX, Thu Vu G, Hoang Nguyen L, Tuan Le Nguyen A, Thanh Tran T, Thanh Nguyen B, et al. Cost-of-illness and the health-related quality of life of patients in the dengue fever outbreak in Hanoi in 2017. Int J Environ Res Public Health. 2018;15:1174. 10.3390/ijerph15061174 29874790 PMC6025163

[13] Suaya JA, Chantha N, Huy R, Sah BK, Moh-Seng C, Socheat D, et al. Clinical characterization, diagnosis and socioeconomic impact of hospitalized dengue in Cambodia [cited 2020 Feb 18]. https://apps.who.int/iris/handle/10665/170966

[14] Armien B, Suaya JA, Quiroz E, Sah BK, Bayard V, Marchena L, et al. Clinical characteristics and national economic cost of the 2005 dengue epidemic in Panama. Am J Trop Med Hyg. 2008;79:364–71. 10.4269/ajtmh.2008.79.364 18784227

[15] Wu S-JL, Grouard-Vogel G, Sun W, Mascola JR, Brachtel E, Putvatana R, et al. Human skin Langerhans cells are targets of dengue virus infection. Nat Med. 2000;6:816–20. 10.1038/77553 10888933

