# Supplementary material for: Health-Related Quality of Life after Dengue Fever, Morelos, Mexico, 2016–2017
Source: Emerg Infect Dis. 2020 Apr;26(4):751–5. doi: 10.3201/eid2604.190729 (PMC7101125; doi:10.3201/eid2604.190729)
Supplement: Appendix — Additional methods and results for study of health-related quality of life after dengue fever, Morelos, Mexico, 2016–2017. [file 19-0729-Techapp-s1.pdf]

Article DOI: https://doi.org/10.3201/eid2604.190729

Health-Related Quality of Life after Dengue Fever, Morelos, Mexico, 2016–2017

Appendix

Additional Context

Dengue fever has become the most important arbovirus disease worldwide due to a rapid spread in the past 50 years (*1–5*). More than half of the world population is living in high risk areas (*5–8*) and a dengue transmission occurs in at least 140 countries worldwide (*9*). Currently, the World Health Organization (WHO) recognizes dengue as one of the top ten threats of global health in 2019 (*10*). Estimates of the global distribution of dengue incidence are heterogeneous. Combining various data sources, including surveillance records, cohort studies, population, and several covariates in a formal model, researchers estimated »390 million annual dengue virus (DENV) infections (95% CI 284–528). Of these, »96 million infections (95% CI 67–136) showed clinical symptoms (*11*). The majority of these clinical DENV infections go unreported or misdiagnosed (*12–19*). Other studies have estimated 50 to 100 million symptomatic DENV infections annually (*9*,*20*), with the most recent estimate at 105 million dengue episodes (*21*) and 40,500 deaths (*22*). Despite variation in the specific estimates of symptomatic dengue episodes, there is vast agreement that dengue is a serious threat to public health systems in most tropical and subtropical countries globally (*8*,*10*,*23*).

Rigorous, comparable estimates of disease burden are important to track health progress, evaluate prevention and control technologies, and define research priorities (*24*,*25*). Several researchers have attempted to estimate the disease burden of dengue (*11*,*15*,*26–34*). But in addition to differences in how symptomatic cases are estimated, there has been heterogeneity in estimating the severity of the disease and sequelae (*9*,*34–36*). In addition to the increase in disease burden, recent studies suggest that symptoms in a DENV infection can go beyond the acute self-limiting febrile phase, and present persistent symptoms in some patients, including fatigue, depression, or weight loss (*28*,*37–45*). Only a few studies investigated the health-related

quality of life (HRQOL) for dengue patients (*37,46,47*). Most studies on the disease burden of dengue, including those that have examined HRQOL in dengue patients, only consider the febrile phase of the illness, despite persistence being acknowledged by WHO since 1997 (*48*), most likely leading to a substantial underestimation of long-term disease burden. To improve current estimations of the disease burden and corresponding economic burden of dengue, studies should take both the febrile and the convalescence phases into consideration (*2,28,49*).

Materials and methods

Sample definition

Participants with dengue were recruited from inpatient and outpatient facilities in 2016–2017 in the state of Morelos, Mexico (*50*). Inclusion criteria were having >18 years of age, the presence of fever between 48 and 144 hours, laboratory-confirmed dengue, a permanent residence, and the availability of a landline telephone. Laboratory confirmation followed the national health secretary's algorithm and guidelines. Patients with cognitive impairment, psychiatric diagnoses, specific chronic diseases (HIV, type 1 diabetes mellitus, multiple sclerosis, lupus and other autoimmune diseases, fibromyalgia, myasthenia, cancers), and pregnancy at recruitment or during the study period were excluded. Of 438 potentially eligible patients, 141 met the inclusion criteria. Of these, 83 had lab-diagnosed dengue. Four patients were excluded during the study because they got pregnant, were unreachable, or voluntarily left the study. The final sample included 79 lab-confirmed dengue patients (Appendix Table 1).

An exhaustive review study by Gómez-Dantés et al. (*29*) on the epidemiology of dengue in Mexico found the incidence of DF and DHF peaked between 10–20, and 15–29 years of age, respectively. However, our sample included only adults ≥18 years of age, for two reasons. First, our study design included follow-up phone calls in which patients had to respond a standardize questionnaire to assess quality of life. While there is a version of EQ-5D for youth (EQ-5D-Y where the lower age limit is 4 years of age), there are no value sets for children and adults (health states are valued differently), and child participants require an adult to respond as a proxy for child's perceived health. This imposes several logistical difficulties for the follow-up. Second, there are several childhood diseases in Mexico that have similar symptoms to dengue, which

would have resulted in a larger number of recruited patients which would have been later confirmed as non-dengue, increasing study costs unnecessarily.

Participants were surveyed at various points in time, from the febrile phase to 1 month after the onset of fever. Follow-up was continued if the patient had persistent symptoms for up to 6 months after the onset of fever (Appendix Table 2). If a participant did not show dengue symptoms during the follow-up interview, she/he was not interviewed in the next round (i.e., the sample size became smaller in time). Estimates of HRQOL thus were limited to patients with persistent symptoms.

Quantifying HRQOL

Patients' HRQOL was measured with an adapted version of the EQ-5D-3L (Spanish version for Mexico) (*51*), including the use of visual analog scale (EQ-VAS) to estimate self-reported health status, which was part of a larger, standardized, structured questionnaire to characterize patients' sociodemographic status and health. The minor adaptation to the EQ-5D-3L consisted in changing two terms of the original questionnaire that are not commonly used in rural Mexico (*lavarme* was changed to *bañarme* and *actividades cotidianas* was changed to *actividades de todos los días*). The EQ-5D-3L questionnaire collects information of the patient's quality of life in five health domains or dimensions: mobility, self-care, usual activities, pain/discomfort, and anxiety/depression. Quality of life was also measured using the EQ-VAS scale, ranging from 0, representing the worse health status, to 100 representing the best health status. Participants used the EQ-VAS scale to represent current health status. We divided time into day ranges, "0 to 6 days," "7 to 15 days," "16 to 30 days," "31 to 60 days," "61 to 120 days," and "121 to 180 days" because not all participants could respond the questionnaires on the exact same days. These day ranges were chosen based on the approximate clinical course of dengue (0–6 and 7–15 are approximately the acute febrile and convalescent phases respectively) and based on previous studies (*37,46*). This choice also guaranteed a sufficient number of participants with completed surveys for each period.

Appendix Table 3 shows the results of health dimensions for the whole study period to complete the already presented data. A large proportion of patients with persistent symptoms (therefore stayed in the study) presented problems mainly in the dimensions pain/discomfort,"

“usual activities,” and “mobility in the time ranges 31 to 60 days,” “61 to 120 days,” and “121 to 180 days” after fever-onset.

To create a single EQ-5D-3L index value for the HRQOL of each patient, the Latin American value set from Zarate et al. (*52*) was used for weighting (Appendix Table 4). To date, there is no specific value set for Mexico available. The value set we used allowed the calculation of 243 different health states, including a social preference weight. The score ranged from 0 to 1 (0 represents the worst and 1 the best health state possible). Participants rated their HRQOL before the onset of dengue during the first interview; this rating was used as a reference value (i.e., baseline) for the description of patient’s HRQOL, and we tracked changes in HRQOL over time.

Data Analysis

We analyzed patients’ longitudinal data using survival analysis and Cox regressions. For those analyses, the development of a time and status variable is necessary. To create the time variable, we defined recovery as reaching the baseline value of HRQOL and calculated the time of each patient from onset of symptoms to recovery. Within the status variable, censored cases (those who did not reach their baseline value) and non-censored cases were classified. We estimated changes in recovery time of HRQOL for different subgroups using the Kaplan-Meier method with the log-rank test statistic. We tested for proportionality to ensure survival analysis was appropriate. As the survival curves show (Appendix Figure), a slower recovery times is found for hospitalized compared to ambulatory participants after 30 days ($p = 0.012$), for participants with severe symptoms compared to participants without severe symptoms after 20 days ($p = 0.001$), for participants with $\geq$1 warning signs compared to participants without warning signs after 15 days ($p<0.001$) and, last for participants with a higher education had a faster recovery of HRQOL than patients with less education ($p<0.001$).

Finally, we identified significant predictors of HRQOL using Cox regression. We chose relevant variables for the Cox regression based on the scientific literature and the group’s clinical expertise in dengue. Based on the size of the study population, a limited number of variables have been added to the model. We checked for multicollinearity using variance inflation factor and the correlation matrix. Significance was defined at $\alpha = 0.05$. We used IBM SPSS Statistics 21 software.

Extended Discussion

The results suggest that dengue fever comes along with a significant reduction of HRQOL beyond the acute febrile phase of disease, consistent with previous research of persistent symptoms. Particularly the dimensions of mobility, pain, and usual activities were the most affected in the first 30 days of the dengue fever episode. These results seem plausible considering the symptoms of the disease. For example, a recent study by Tran et al. (*53*) about dengue patients in Hanoi also suggested that the most affected dimensions of HRQOL were mobility (62.3% of participants), usual activities (64.4% of participants) and self-care (71.8% of participants). The proportion of patients with pain was much lower in the Hanoi study than in our study (32.2% of participants). Tran et al. (*53*) measured quality of life at a single point in time; in contrast we took several measurements during the febrile phase of the disease and after. Another study by Lum et al. (*46*) described the HRQOL in dengue patients in Malaysia in the first 20 days of disease. They found the most affected dimensions were usual activities, pain, cognition, and interpersonal activities. For example, 94% of the study population reported problems in the dimension of pain, consistent with our study, although we used a different instrument to assess HRQOL.

Anxiety and depression have been previously described as symptoms among dengue patients during the febrile phase (*54*). Our study showed that almost 34% of dengue patients had problems with anxiety or depression in the first 6 days of the disease. The frequency of anxiety or depression increased in the following periods up to 40.5%. Other studies have shown even higher rates of anxiety or depression in dengue patients (*54*). One explanation recording to Tran et al. might be the association with increasing media attention on this topic. If increased media attention is a factor for Mexico, it is not clear at this point.

The results of the descriptive health profile showed the percentage of patients that report problems in any of the five dimensions considered by EQ-5D-3L remain stable in patients with persistent symptoms. Our results clearly demonstrated a reduction in HRQOL in the first days of illness. HRQOL increased steadily following the febrile phase, although for some patients, HRQOL had not yet reached baseline value after 6 months. It is relevant to note that because the follow-up was continued only if the patient had persistent symptoms for up to 6 months post

fever-onset, the sample size decreased in time, so direct comparisons of health status may be problematic.

Different results of those measurements of HRQOL of dengue patients exist in the literature. Suaya et al. (*55*) investigated the HRQOL of children with dengue in Cambodia and show a stronger reduction of HRQOL compared to our findings (average EQ-VAS of seven). This may be partially explained because Suaya et al.'s study population were children aged 0 to 14 years old. With a median EQ-VAS of 10 for inpatients and 20 for outpatients, Martelli et al. (*37*) observed a lower HRQOL of dengue patients (adults) in Brazil. Our findings are supported by the results from Armien et al. (*56*). In their study, the median of EQ-VAS was 35.2 for children and 31.9 for adults. All these studies used the EQ-VAS to describe the HRQOL of dengue patients. By using EQ-VAS plus the index score more information was generated. The same pattern emerged from both parts of the EQ-5D-3L and were used to support the results. Just one study identified used the index score in dengue patients. This study among dengue patients (adults) in Hanoi showed a higher index (0.66) compared to our findings (*53*).

Consistent with previous studies (*41,44,57*), we found female gender was associated with persistence of symptoms. There have been heterogenous findings about differences in HRQOL of hospitalized and ambulatory dengue patients. While Martelli et al. (*37*) found evidence for significant differences in those two groups, Tran et al. (*53*) did not. Both studies were cross-sectional (i.e., had only one measurement). We found differences, which seem plausible considering that patients with severe dengue symptoms are commonly hospitalized. These findings are relevant for clinical practice, as they could imply recurrent care for some patients. For patients with persistent symptoms, with at least one severe symptom or warning sign, abdominal pain, persistent vomiting, clinical accumulation of fluids, bleeding in mucosae, lethargy or restlessness, and liver enlargement (*1*), should receive special attention in clinic because of their increased risk of long-term reduction of their HRQOL.

By using a Cox regression we identified several factors that were associated with recovery of HRQOL. Previous research has shown that female gender and older age are significantly associated with severity of dengue episodes (*41,44,57*) and that a higher educational level of dengue patients can be identified as a protective factor (*53,58*). Our findings agree with previous research, but we failed to find a statistically significant correlation between age of the

participant and persistence of symptoms, perhaps due to the relatively small sample size in our study. Discussions about plausible mechanisms to explain those findings are related to the health status and income of patients. Regarding our results, it should be noted that despite the significant p-value, the range of the confidence interval showed a lot of variation in the relative risk of recovering from dengue. Due to that, the effect of gender and educational level on the recovery of HRQOL might be very small.

Our study indicates specific skin symptoms as factors positively affecting the increase in HRQOL over time. This might be explained by the fact that skin symptoms, such as a painful and itchy rash, are linked to the interaction of the virus with the host cells that release various chemical mediators and initiate an immune response (*59*). It is thus assumed that the rash in dengue fever may not be released by the virus, but due to proteins of an intact immune system. Patients with a skin rash do not show more complications or a poorer state of health during the illness than patients without skin rash (*60*). This indicates that a better understanding of skin symptoms in dengue fever is needed to improve diagnosis and treatment (*61*).

The present study has some limitations. The study population includes only adults. Research findings demonstrate differences between adults and children regarding the pattern of complications (*62,63*). Because of that, the investigation of the HRQOL in children with dengue fever would be important for both clinical practice, as well as for economic impact. Our findings cannot generalize to the whole Mexican population of dengue patients. Due to our study's restriction to adult patients, our results cannot be directly compared to those other studies with participants in wider age ranges. Further, some variables that may have a significant role in determining HRQOL, such as socio-economic factors, were not included in the questionnaires and could therefore not be investigated. Furthermore, the altitude, for example could not be examined because the study population was homogenous on this characteristic; also, no data were available on prior dengue infections. Although the EQ-5D-3L is a validated instrument, limitations such as ceiling- and floor effects, especially in the descriptive part of the EQ-5D, are possible. Another limitation is a possible recall bias when assessing the baseline value of HRQOL after the onset of the disease. Other potential sources of bias include response-shift, recalibration-response-shift, and reconceptualization-response-shift biases (*64*).

The use of value sets is an important factor in analyzing data of HRQOL. Because there is no Mexican value set available, we used a set based on Latin-Americans living in the U.S. This population may not be representative of the Mexican population. Last, our study population is relatively small and might not be representative of all adult dengue fever patients in Mexico.

This study highlights the relevance of public health research on dengue fever, especially in terms of patient's HRQOL and the persistent symptoms of the disease. The findings of this study are relevant for clinical practice and also for health services research. To accurately estimate the disease and economic burden of dengue, it is important to understand how HRQOL varies in time, beyond the febrile phase of the illness. Such a comprehensive understanding of burden of disease should inform assessments of the disease burden and strategies to control and prevent the disease.

References

1. World Health Organization. Dengue: guidelines for diagnosis, treatment, prevention and control. New edition. 2009 [cited 2020 Feb 18]. http://apps.who.int/iris/bitstream/10665/44188/1/9789241547871_eng.pdf

2. Simmons CP, Farrar JJ, Nguyen V, Wills B. Dengue. N Engl J Med. 2012;366:1423–32. PubMed https://doi.org/10.1056/NEJMra1110265

3. Gubler DJ. Dengue viruses: their evolution, history, and emergence as a global public health problem. In: Gubler D, Ooi EE, Vasudevan S, Farrar J, editors. Dengue and dengue hemorrhagic fever. 2nd ed. Wallingford (UK): CAB International; 2014. p. 1–29.

4. Messina JP, Brady OJ, Scott TW, Zou C, Pigott DM, Duda KA, et al. Global spread of dengue virus types: mapping the 70 year history. Trends Microbiol. 2014;22:138–46. PubMed https://doi.org/10.1016/j.tim.2013.12.011

5. Messina JP, Brady OJ, Golding N, Kraemer MUG, Wint GRW, Ray SE, et al. The current and future global distribution and population at risk of dengue. Nat Microbiol. 2019;4:1508–15. PubMed https://doi.org/10.1038/s41564-019-0476-8

6. Kraemer MUG, Sinka ME, Duda KA, Mylne AQN, Shearer FM, Barker CM, et al. The global distribution of the arbovirus vectors *Aedes aegypti* and *Ae. albopictus.* eLife. 2015;4:e08347. PubMed http://doi.org/10.7554/eLife.08347

7. Kraemer MUG, Reiner RC, Brady OJ, Messina JP, Gilbert M, Pigott DM, et al. Past and future spread of the arbovirus vectors *Aedes aegypti* and *Aedes albopictus*. Nat Microbiol. 2019;4:854–63. https://doi.org/10.1038/s41564-019-0376-y

8. Castro MC, Wilson ME, Bloom DE. Disease and economic burdens of dengue. Lancet Infect Dis. 2017;17:e70–8. PubMed http://doi.org/10.1016/S1473-3099(16)30545-X

9. Stanaway JD, Shepard DS, Undurraga EA, Halasa YA, Coffeng LE, Brady OJ, et al. The global burden of dengue: an analysis from the Global Burden of Disease Study 2013. Lancet Infect Dis. 2016;16:712–23. PubMed https://doi.org/10.1016/S1473-3099(16)00026-8

10. World Health Organization. Ten threats to global health in 2019 [cited 2020 Feb 18]. https://www.who.int/emergencies/ten-threats-to-global-health-in-2019

11. Bhatt S, Gething PW, Brady OJ, Messina JP, Farlow AW, Moyes CL, et al. The global distribution and burden of dengue. Nature. 2013;496:504–7. PubMedhttps://doi.org/10.1038/nature12060

12. Sarti E, L'Azou M, Mercado M, Kuri P, Siqueira JB Jr, Solis E, et al. A comparative study on active and passive epidemiological surveillance for dengue in five countries of Latin America. Int J Infect Dis. 2016;44:44–9. PubMed https://doi.org/10.1016/j.ijid.2016.01.015

13. Silva MM, Rodrigues MS, Paploski IA, Kikuti M, Kasper AM, Cruz JS, et al. Accuracy of dengue reporting by national surveillance system, Brazil. Emerg Infect Dis. 2016;22:336–9. PubMed https://doi.org/10.3201/eid2202.150495

14. Undurraga EA, Halasa YA, Shepard DS. Use of expansion factors to estimate the burden of dengue in Southeast Asia: a systematic analysis. PLoS Negl Trop Dis. 2013;7:e2056. PubMed https://doi.org/10.1371/journal.pntd.0002056

15. Undurraga EA, Betancourt-Cravioto M, Ramos-Castañeda J, Martínez-Vega R, Méndez-Galván J, Gubler DJ, et al. Economic and disease burden of dengue in Mexico. PLoS Negl Trop Dis. 2015;9:e0003547. PubMed https://doi.org/10.1371/journal.pntd.0003547

16. Sharp TM, Moreira R, Soares MJ, Miguel da Costa L, Mann J, DeLorey M, et al. Underrecognition of dengue during 2013 epidemic in Luanda, Angola. Emerg Infect Dis. 2015;21:1311–6. PubMed https://doi.org/10.3201/eid2108.150368

17. Standish K, Kuan G, Avilés W, Balmaseda A, Harris E. High dengue case capture rate in four years of a cohort study in Nicaragua compared to national surveillance data. PLoS Negl Trop Dis. 2010;4:e633. PubMed https://doi.org/10.1371/journal.pntd.0000633

18. Wichmann O, Yoon IK, Vong S, Limkittikul K, Gibbons RV, Mammen MP, et al. Dengue in Thailand and Cambodia: an assessment of the degree of underrecognized disease burden based on reported cases. PLoS Negl Trop Dis. 2011;5:e996. PubMed https://doi.org/10.1371/journal.pntd.0000996

19. Toan NT, Rossi S, Prisco G, Nante N, Viviani S. Dengue epidemiology in selected endemic countries: factors influencing expansion factors as estimates of underreporting. Trop Med Int Health. 2015;20:840–63. PubMed https://doi.org/10.1111/tmi.12498

20. Rigau-Pérez JG, Clark GG, Gubler DJ, Reiter P, Sanders EJ, Vorndam AV. Dengue and dengue haemorrhagic fever. Lancet. 1998;352:971–7. PubMed https://doi.org/10.1016/S0140-6736(97)12483-7

21. James SL, Abate D, Abate KH, Abay SM, Abbafati C, Abbasi N, et al.; GBD 2017 Disease and Injury Incidence and Prevalence Collaborators. Global, regional, and national incidence, prevalence, and years lived with disability for 354 diseases and injuries for 195 countries and territories, 1990–2017: a systematic analysis for the Global Burden of Disease Study 2017. Lancet. 2018;392:1789–858. PubMed https://doi.org/10.1016/S0140-6736(18)32279-7

22. Roth GA, Abate D, Abate KH, Abay SM, Abbafati C, Abbasi N, et al.; GBD 2017 Causes of Death Collaborators. Global, regional, and national age-sex-specific mortality for 282 causes of death in 195 countries and territories, 1980–2017: a systematic analysis for the Global Burden of Disease Study 2017. Lancet. 2018;392:1736–88. PubMed https://doi.org/10.1016/S0140-6736(18)32203-7

23. Herricks JR, Hotez PJ, Wanga V, Coffeng LE, Haagsma JA, Basáñez M-G, et al. The global burden of disease study 2013: what does it mean for the NTDs? PLoS Negl Trop Dis. 2017;11:e0005424. PubMed https://doi.org/10.1371/journal.pntd.0005424

24. Chan M, Kazatchkine M, Lob-Levyt J, Obaid T, Schweizer J, Sidibe M, et al. Meeting the demand for results and accountability: a call for action on health data from eight global health agencies. PLoS Med. 2010;7:e1000223. PubMed https://doi.org/10.1371/journal.pmed.1000223

25. Frieden TR. Government's role in protecting health and safety. N Engl J Med. 2013;368:1857–9. PubMed https://doi.org/10.1056/NEJMp1303819

26. Zubieta-Zavala A, Salinas-Escudero G, Ramírez-Chávez A, García-Valladares L, López-Cervantes M, López Yescas JG, et al. Calculation of the average cost per case of dengue fever in Mexico using

a micro-costing approach. PLoS Negl Trop Dis. 2016;10:e0004897. PubMed https://doi.org/10.1371/journal.pntd.0004897

27. Zubieta-Zavala A, López-Cervantes M, Salinas-Escudero G, Ramírez-Chávez A, Castañeda JR, Hernández-Gaytán SI, et al. Economic impact of dengue in Mexico considering reported cases for 2012 to 2016. PLoS Negl Trop Dis. 2018;12:e0006938. PubMed https://doi.org/10.1371/journal.pntd.0006938

28. Tiga DC, Undurraga EA, Ramos-Castañeda J, Martínez-Vega RA, Tschampl CA, Shepard DS. Persistent symptoms of dengue: estimates of the incremental disease and economic burden in Mexico. Am J Trop Med Hyg. 2016;94:1085–9. PubMed https://doi.org/10.4269/ajtmh.15-0896

29. Dantés HG, Farfán-Ale JA, Sarti E. Epidemiological trends of dengue disease in Mexico (2000–2011): a systematic literature search and analysis. PLoS Negl Trop Dis. 2014;8:e3158. PubMed https://doi.org/10.1371/journal.pntd.0003158

30. Shepard DS, Coudeville L, Halasa YA, Zambrano B, Dayan GH. Economic impact of dengue illness in the Americas. Am J Trop Med Hyg. 2011;84:200–7. PubMed https://doi.org/10.4269/ajtmh.2011.10-0503

31. Shepard DS, Undurraga EA, Halasa YA. Economic and disease burden of dengue in Southeast Asia. PLoS Negl Trop Dis. 2013;7:e2055. PubMed https://doi.org/10.1371/journal.pntd.0002055

32. Shepard DS, Undurraga EA, Halasa YA, Stanaway JD. The global economic burden of dengue: a systematic analysis. Lancet Infect Dis. 2016;16:935–41. PubMed https://doi.org/10.1016/S1473-3099(16)00146-8

33. Brady OJ, Gething PW, Bhatt S, Messina JP, Brownstein JS, Hoen AG, et al. Refining the global spatial limits of dengue virus transmission by evidence-based consensus. PLoS Negl Trop Dis. 2012;6:e1760. PubMed https://doi.org/10.1371/journal.pntd.0001760

34. Zeng W, Halasa-Rappel YA, Durand L, Coudeville L, Shepard DS. Impact of a nonfatal dengue episode on disability-adjusted life Years: a systematic analysis. Am J Trop Med Hyg. 2018;99:1458–65. PubMed https://doi.org/10.4269/ajtmh.18-0309

35. Meltzer MI, Rigau-Pérez JG, Clark GG, Reiter P, Gubler DJ. Using disability-adjusted life years to assess the economic impact of dengue in Puerto Rico: 1984–1994. Am J Trop Med Hyg. 1998;59:265–71. PubMed https://doi.org/10.4269/ajtmh.1998.59.265

36. Hung TM, Clapham HE, Bettis AA, Cuong HQ, Thwaites GE, Wills BA, et al. The estimates of the health and economic burden of dengue in Vietnam. Trends Parasitol. 2018;34:904–18. PubMed https://doi.org/10.1016/j.pt.2018.07.007

37. Martelli CMT, Nascimento NE, Suaya JA, Siqueira JB Jr, Souza WV, Turchi MD, et al. Quality of life among adults with confirmed dengue in Brazil. Am J Trop Med Hyg. 2011;85:732–8. PubMed https://doi.org/10.4269/ajtmh.2011.11-0067

38. Teixeira LAS, Nogueira FPDS, Nascentes GAN. Prospective study of patients with persistent symptoms of dengue in Brazil. Rev Inst Med Trop São Paulo. 2017;59:e65. PubMed https://doi.org/10.1590/s1678-9946201759065

39. Teixeira LA, Lopes JSM, Martins AGD, Campos FAB, Miranzi SS, Nascentes GAN. [Persistence of dengue symptoms in patients in Uberaba, Minas Gerais State, Brazil]. Cad Saude Publica. 2010;26:624–30. PubMed https://doi.org/10.1590/S0102-311X2010000300019

40. Seet RCS, Quek AML, Lim ECH. Post-infectious fatigue syndrome in dengue infection. J Clin Virol. 2007;38:1–6. PubMed https://doi.org/10.1016/j.jcv.2006.10.011

41. García G, González N, Pérez AB, Sierra B, Aguirre E, Rizo D, et al. Long-term persistence of clinical symptoms in dengue-infected persons and its association with immunological disorders. Int J Infect Dis. 2011;15:e38–43. PubMed https://doi.org/10.1016/j.ijid.2010.09.008

42. López Barroso R, Deulofeu Betancourt I, Fayad Saeta Y, Macias Navarro MM. Convalecencia de mujeres que sufrieron dengue serotipo 3 durante el embarazo. Rev Cubana Med Trop. 2011;63:206–10. PubMed

43. Tristão-Sá R, Kubelka CF, Zandonade E, Zagne SMO, Rocha NS, Zagne LO, et al. Clinical and hepatic evaluation in adult dengue patients: a prospective two-month cohort study. Rev Soc Bras Med Trop. 2012;45:675–81. PubMed https://doi.org/10.1590/S0037-86822012000600004

44. Halsey ES, Williams M, Laguna-Torres VA, Vilcarromero S, Ocaña V, Kochel TJ, et al. Occurrence and correlates of symptom persistence following acute dengue fever in Peru. Am J Trop Med Hyg. 2014;90:449–56. PubMed https://doi.org/10.4269/ajtmh.13-0544

45. Luengas LL, Tiga DC, Herrera VM, Villar-Centeno LÁ. Characterization of the health condition of people convalescing from a dengue episode. Biomedica. 2015;36:89–97. PubMed http://doi.org/10.7705/biomedica.v36i0.3019

46. Lum LCS, Suaya JA, Tan LH, Sah BK, Shepard DS. Quality of life of dengue patients. Am J Trop Med Hyg. 2008;78:862–7. PubMed https://doi.org/10.4269/ajtmh.2008.78.862

47. Liu E, Vu D, Boothroyd D, Ndenga B, Onyango W, Okuta V, et al. Evaluation of the health-related quality of life of children with dengue and malaria in western Kenya. Open Forum Infect Dis. 2016;3(suppl_1):591.

48. World Health Organization. Dengue haemorrhagic fever: diagnosis, treatment, prevention and control. 1997 [cited 2020 Feb 18]. http://www.who.int/csr/resources/publications/dengue/Denguepublication/en/

49. Shepard DS, Undurraga EA, Betancourt-Cravioto M, Guzmán MG, Halstead SB, Harris E, et al. Approaches to refining estimates of global burden and economics of dengue. PLoS Negl Trop Dis. 2014;8:e3306. PubMed https://doi.org/10.1371/journal.pntd.0003306

50. Tiga-Loza DC, Martínez-Vega RA, Undurraga EA, Tschampl CA, Shepard DS, Ramos-Castañeda J. Persistence of symptoms in dengue patients: a clinical cohort study. Trans R Soc Trop Med Hyg. 2020. In press,

51. EuroQol Group. EuroQol—a new facility for the measurement of health-related quality of life. Health Policy. 1990;16:199–208. PubMed https://doi.org/10.1016/0168-8510(90)90421-9

52. Zarate V, Kind P, Chuang LH. Hispanic valuation of the EQ-5D health states: a social value set for Latin Americans. Value Health. 2008;11:1170–7. PubMed https://doi.org/10.1111/j.1524-4733.2008.00349.x

53. Tran BX, Thu Vu G, Hoang Nguyen L, Tuan Le Nguyen A, Thanh Tran T, Thanh Nguyen B, et al. Cost-of-illness and the health-related quality of life of patients in the dengue fever outbreak in Hanoi in 2017. Int J Environ Res Public Health. 2018;15:1174. PubMed https://doi.org/10.3390/ijerph15061174

54. Hashmi AM, Butt Z, Idrees Z, Niazi M, Yousaf Z, Haider SF, et al. Anxiety and depression symptoms in patients with dengue fever and their correlation with symptom severity. Int J Psychiatry Med. 2012;44:199–210. PubMed https://doi.org/10.2190/PM.44.3.b

55. Suaya JA, Chantha N, Huy R, Sah BK, Moh-Seng C, Socheat D, et al. Clinical characterization, diagnosis and socioeconomic impact of hospitalized dengue in Cambodia. Dengue Bulletin. 2010;34:89–103.

56. Armien B, Suaya JA, Quiroz E, Sah BK, Bayard V, Marchena L, et al. Clinical characteristics and national economic cost of the 2005 dengue epidemic in Panama. Am J Trop Med Hyg. 2008;79:364–71. PubMed https://doi.org/10.4269/ajtmh.2008.79.364

57. da Silva NS, Undurraga EA, da Silva Ferreira ER, Estofolete CF, Nogueira ML. Clinical, laboratory, and demographic determinants of hospitalization due to dengue in 7613 patients: A retrospective study based on hierarchical models. Acta Trop. 2018;177:25–31. PubMed http://doi.org/10.1016/j.actatropica.2017.09.025

58. da Silva NS, Undurraga EA, Verro AT, Nogueira ML. Comparison between the traditional (1997) and revised (2009) WHO classifications of dengue disease: a retrospective study of 30 670 patients. Trop Med Int Health. 2018;23:1282–93. PubMed https://doi.org/10.1111/tmi.13155

59. de Andino RM, Botet MV, Gubler DJ, García C, Laboy E, Espada F, et al. The absence of dengue virus in the skin lesions of dengue fever. Int J Dermatol. 1985;24:48–51. PubMed https://doi.org/10.1111/j.1365-4362.1985.tb05360.x

60. Huang H-W, Tseng H-C, Lee C-H, Chuang H-Y, Lin S-H. Clinical significance of skin rash in dengue fever: a focus on discomfort, complications, and disease outcome. Asian Pac J Trop Med. 2016;9:713–8. PubMed https://doi.org/10.1016/j.apjtm.2016.05.013

61. Azfar NA, Malik LM, Jamil A, Jahangir M, Tirmizi N, Majid A, et al. Cutaneous manifestations in patients of dengue fever. Journal of Pakistan Association of Dermatologists. 2012;22:320–4.

62. Trung DT, Thao TT, Dung NM, Ngoc TV, Hien TT, Chau NV, et al. Clinical features of dengue in a large Vietnamese cohort: intrinsically lower platelet counts and greater risk for bleeding in adults than children. PLoS Negl Trop Dis. 2012;6:e1679. PubMed https://doi.org/10.1371/journal.pntd.0001679

63. Namvongsa V, Sirivichayakul C, Songsithichok S, Chanthavanich P, Chokejindachai W, Sitcharungsi R. Differences in clinical features between children and adults with dengue hemorrhagic fever/dengue shock syndrome. Southeast Asian J Trop Med Public Health. 2013;44:772–9. PubMed

64. Blome C, Augustin M. Measuring change in quality of life: bias in prospective and retrospective evaluation. Value Health. 2015;18:110–5. PubMed https://doi.org/10.1016/j.jval.2014.10.007

Appendix Table 1. Descriptive statistics for final sample of lab-confirmed dengue patients in Morelos, Mexico 2016–2017

Characteristic	Hospitalized, no. (%)	Ambulatory, no. (%)
Age, y, n = 79		
18–29	10 (32.3)	21 (67.7)
30–49	13 (35.1)	24 (64.9)
≥50	7 (63.6)	4 (36.4)
Gender, n = 79		
F	14 (31.1)	31 (68.9)
M	16 (47.1)	18 (52.9)
Education, n = 73		
Primary/secondary	20 (37.0)	34 (63.0)
High-school or higher	6 (31.6)	13 (68.4)
Occupation, n = 78		
Student	0	5 (100.0)
In employment/with income	16 (41.0)	23 (59.0)
Not in employment/no income	15 (41.2)	20 (58.8)
Persistence of symptoms, n = 79		
Symptoms ≤30 d	9 (25.7)	26 (74.3)
Symptoms >30 d	21 (47.7)	23 (52.3)
Severity of symptoms, n = 78		
No severe symptoms	9 (22.5)	31 (77.5)
Severe symptoms, at least 1	20 (52.6)	18 (47.4)
Warning signs, n = 79		
Without warning sings	0	21 (100.0)
With warning signs	30 (51.7)	28 (48.3)

Appendix Table 2. Sampling points of the data collection

Month*	Month (1)																											
Week†	Week (1)							Week (2)							Week (3)							Week (4)						
Day	1	2	3	4	5	6	7	8	9	10	11	12	13	14	15	16	17	18	19	20	21	22	23	24	25	26	27	28
Activity‡				Visit (1)					Call (1)				Call (2)					Call (3)							Call (4)			

Month*			Month (2)					Month (3)				Month (4)				Month (5)				Month (6)	
Week†	5	6	7	8	9	10	11	12	13	14	15	16	17	18	19	20	21	22	23	24	25
Activity‡	Visit (2)		Call (5)		Call (6)			Visit (3)§			Call (7)			Call (8)			Call (9)			Call (10)	

*Month number (1) when subject was recruited.
†Week number.
‡Activity number. Subject’s home visit (visit) or subjects contacted by telephone call (call).
§In case of fever persistence.

Appendix Table 3. Share of 79 lab-confirmed dengue patients (>18 y of age) reporting some or extreme problems with health-related quality of life dimensions (adapted EQ-5D-3L) of the whole study period

	Days						
Dimensions EQ-5D-3L	Baseline, no. (%)	1–6, no. (%)	7–15, no. (%)	16 −30, no. (%)	31–60, no. (%)	61–120, no. (%)*	121–180, no. (%)*
Mobility	1 (1.3)	63 (79.7)	57 (80.3)	59 (79.7)	53 (85.5)	28 (84.8)	6 (85.7)
Self-care	0	43 (54.4)	39 (54.9)	42 (56.8)	40 (64.5)	22 (66.7)	5 (71.4)
Usual activities	2 (2.5)	69 (87.3)	65 (91.5)	65 (87.8)	57 (91.9)	32 (97)	7 (100)
Pain / discomfort	2 (2.5)	73 (92.4)	66 (93)	63 (85.1)	59 (95.2)	30 (90.9)	7 (100)
Anxiety/depression	4 (5.1)	27 (34.2)	28 (39.4)	30 (40.5)	25 (40.3)	16 (48.5)	4 (57.1)
Total (n)	77	79	71	74	62	33	7

*The proportion of patients presenting some or extreme problems with health-related quality of life dimensions 1 month after the onset of fever or after 1 month, only includes patients who reported symptoms. Patients who reported no symptoms were no longer contacted after the first month and thus did not respond the questionnaire.

Appendix Table 4. N3+X4 model used for calculating the EQ-5D index for each patient with preference weighting*

Parameter	Coefficient	SD	p value
Constant	0.125	0.015	<0.001
M2	0.047	0.012	<0.002
M3	0.290	0.015	<0.003
SC2	0.054	0.012	<0.004
SC3	0.176	0.015	<0.005
Ua2	0.801	0.016	<0.006
Ua3	0.181	0.019	<0.007
Pd2	0.103	0.014	<0.008
Pd3	0.202	0.015	<0.009
Ad2	0.060	0.012	<0.010
Ad3	0.122	0.013	<0.011
N3	0.079	0.018	<0.012
X4	0.074	0.020	<0.013
R^2	0.332		
MAE	0.031		
No (out of 42) >0.05	8		
No (out of 42) >0.10	0		

*Calculations based on Zarate et al. (*52*)

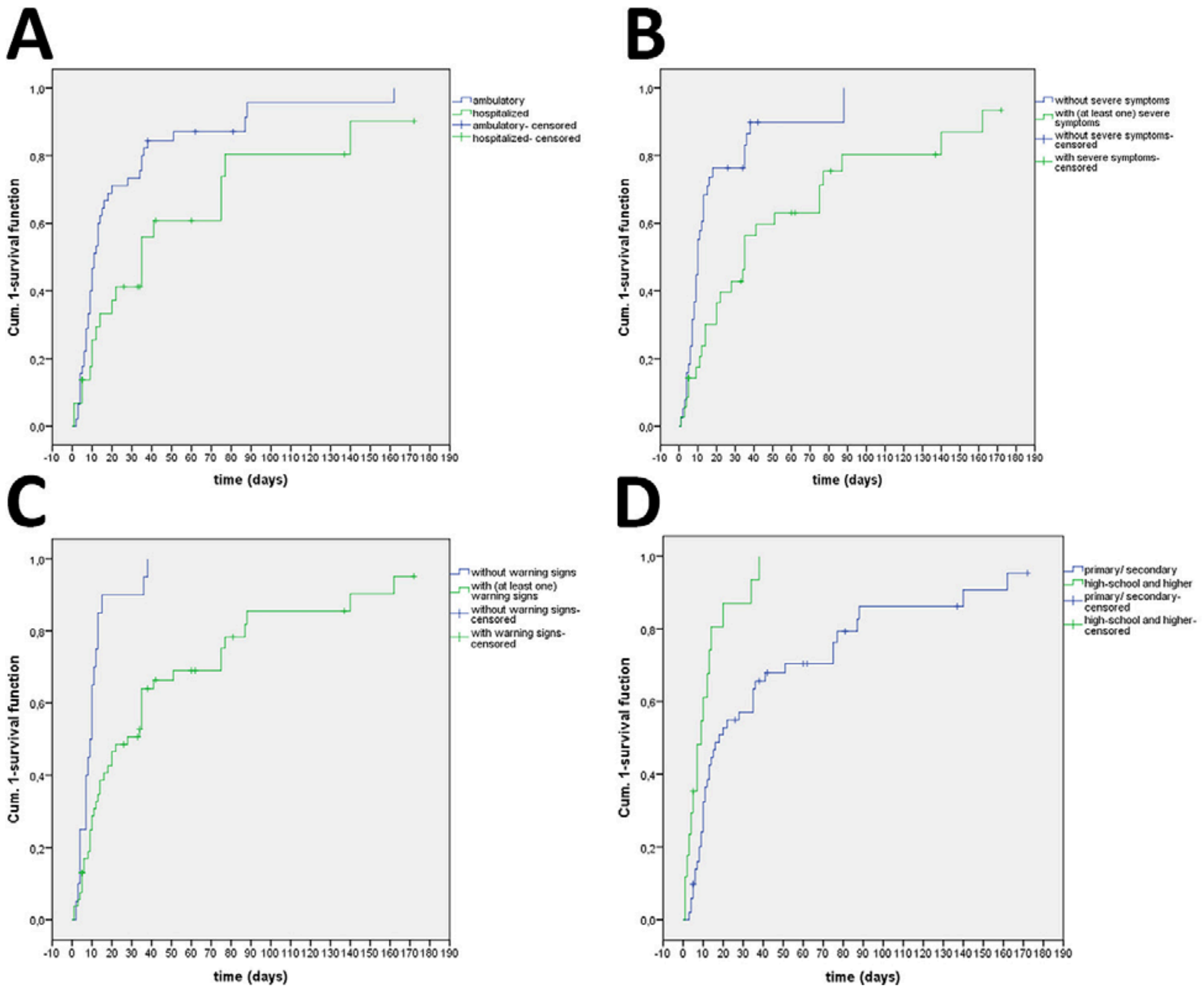

Appendix Figure. Survival curve of lab-confirmed dengue patients in Morelos, Mexico, 2016–2017 comparing (A) ambulatory and hospitalized, (B) with and without severe symptoms, (C) with and without warning signs, and (D) by level of formal education.
